# Characterisation of treatment resistant mood disorder patients in the United Arab Emirates

**DOI:** 10.1007/s00127-025-02878-4

**Published:** 2025-04-03

**Authors:** Danilo Arnone, Emmanuel Stip

**Affiliations:** 1 College of Medicine, Mohammed Bin Rashid University of Medicine and Health Sciences, Dubai Health, Dubai, United Arab Emirates; 2 Present Address: Centre for Affective Disorders, Psychological Medicine, Institute of Psychiatry, Psychology and Neurosciences, King’s College London, London, UK; 3 Present Address: Department of Psychiatry, Montreal University, Centre Hospitalier Universitaire de l’Université de Montréal, Instititut Universitaire en Santé mentale de Montréal, Montreal, QC Canada; 4 Department of Psychiatry, College of Medicine and Health Sciences, United Arab Emirates University, Al-Ain, United Arab Emirates

**Keywords:** Mood disorders, Depression, Treatment resistant depression, United Arab Emirates

## Abstract

There is paucity of mental health epidemiological data from the Middle East. Therein we present detailed clinical characterization of mood disorders patients from a university tertiary clinic established in the United Arab Emirates. The majority of patient experienced depressive and anxiety symptoms largely in the moderate range according to both self-rated and clinician rated scales, accompanied by significant co-morbidities, functional impairment, mild reduction in cognitive function, and a significant ruminative thinking style. These preliminary findings support the need for larger-scale research in the Middle East to address the burden of poor mental health affecting communities.

**Supplementary Information:**

The online version contains supplementary material available at 10.1007/s00127-025-02878-4.

Clinical characteristics of treatment-resistant mood disorders around the world are not well described. This is particularly significant in the Middle East where there is paucity of basic mental health epidemiological data [1]. In the United Arab Emirates (UAE), the most accurate prevalence of mental disorders was acquired in Al Ain, Abu Dhabi Emirate over twenty years ago. In this report, mood disorders figured among the most frequent conditions, women were at much higher lifetime risk of developing depressive disorders compared to men (10.3 vs. 2.8%) and female sex was the strongest vulnerability factor associated with life events, chronic difficulties and a prole of ≥4 children at home (independent of marital status) [2].

Therein is included data from a tertiary level mood disorder service created in 2020 by the United Arab Emirates University at Al Ain Hospital in the Eastern region of the Emirate of Abu Dhabi.

Abu Dhabi Emirate accounts for 84 per cent of the country’s total landmass (https://u.ae/en/about-the-uae/fact-sheet) with a population of approximately 3 million (11 million total UAE population, https://eds.ae/uae-population-stastics-demographic-break-down-emirates-wise/). Al Ain hospital is the largest hospital outside Abu Dhabi capital covering approximately 30% of the territory [3]. Al Ain hospital includes a range of inpatients and outpatients’ psychiatric services including specialised services [3]. The mood disorder clinic was active for three years and anonymised data were collected as part of a quality improvement project (ethics approvals AAHEC − 11-19-029 and MF2058-2022-850).

Diagnostic assessments were based on the Mini International Neuropsychiatry Interview for DSM-IV [4] and patients received ICD-10 diagnostic codes [5]. Level of treatment refractoriness was established according to the Maudsley Staging Method, based on number of treatment failures (including details of adequate dose/length of treatment), use of augmentation strategies and electroconvulsive therapy [6]. Twenty-four patients were assessed (see table 1 and supplementary material for details). Fifteen patients were UAE natives, 2 from Pakistan and 1 from India, South Africa, Egypt, Somalia, Syria, Afghanistan, and Oman. Diagnoses included major depressive disorders (*N* = 21) and bipolar disorders (*N* = 3). Thirteen patients presented with recurrent major depression (N. episodes mode = 3), 6 mild (F33.0), 6 moderate (F33.1), 1 severe without psychotic symptoms (F33.2). Eight experienced a single depressive episode, 1 mild (F32.0), 5 moderate (F32.1) and 2 severe (F32.2). The three bipolar patients were hypomanic (F31.0), in remission (F31.74) and moderately depressed (F31.3). Comorbidities included 10 cases of generalised anxiety disorder (F41.1), 1 borderline personality disorder (F60.3), 4 social phobia (F40.10), 1 agoraphobia (F41.0), 1 specific phobia (F40.218), 1 post-traumatic stress disorder (F43.12), 2 ADHD (F90.9), 2 alcohol abuse (F10.1, F10.21), 1 OCD (F42).

Unipolar major depression was the commonest presentation (87%) and depressive and anxiety symptoms were largely of moderate intensity according to both self- and clinician-rated scales. Co-morbidities commonly included generalised anxiety disorder (42%), and social phobia (17%). Patients failed an average of 3 pharmacological trials, pharmacological augmentation was used in 74% of cases and ECT in 17%. Functional impairment was significant, cognitive function was mildly reduced, thinking style was on average ruminative and emotional and physical maltreatment were present.

This is the first short report detailing the clinical characteristics of treatment resistant patients from the United Arab Emirates. Compared to similar services, sexes were equally represented and age was younger, whilst mean levels of treatment resistance and number of treatment failures were similar e.g [7, 8]. Since the unification of the country in 1971, modern development has been exponential in the UAE with progressive acquisition of western values, technologies and commodities impacting on native way of life, often contrasting traditional social, cultural and religious beliefs. Less traditional individuals living in a rapidly changing society suffer from higher levels of psychopathology [9] which might explain the younger age of patients and an almost equal sex distribution.

In the Middle East feelings of embarrassment and shame and negative societal attitudes towards mental illness (not uncommon in many societies), are amplified by religious beliefs that being mentally unwell can be the result of lack of faith, whereas hardship and sufferance are viewed as divine tests requiring acceptance. Endurance of symptoms is therefore common, similarly to a reluctance to engage in talking therapies [10]. The UAE is not exempt from issues such as stigma, a tendency to cultural and religious interpretation of symptoms, and a preference for traditional and religious healers, preventing timely engagement with mental health services [1]. The noticeable underutilization of psychological therapies although could be associated with limited availability, might also be related to a tendency to attribute mental illness to an external locus of control, discouraging self-exploration to support recovery. It is also possible that psychological treatments, largely developed in the West, would benefit from adaptations or newly developed paradigms that take into consideration not just language differences but also local customs and beliefs. Insufficient insurance coverage for mental illness for expatriates is another barrier limiting access to mental health services as suggested by the predominance of local patients (62%), which is consistent with previous observations [3].

Depression affects 1/10 of the world’s population and is expected to become the first cause of disability by 2030. Gulf Cooperation Countries (GCC) are not immune to the burden of depressive disorders. A strategic public health approach is required in the region to tackle the burden of depression and would benefit from scientific rigour, based on the observation that available literature is scant and lacks scientific depth [11]. In addition, the documented poor adherence to practice guidelines is not conducive to the resolution of mood symptoms, considering that systematicity is important in the treatment of mood disorders. This could be due to the lack of culturally tailored recommendations [12].

In conclusion, although these findings are preliminary, can support generating hypotheses for future, larger-scale research in the GCC region to address the burden of poor mental health affecting communities, particularly the younger generations [11].

**Table 1:** Clinical and demographic characteristics of the sample. See text and supplementary information for explanation and references. MSM: Maudsley Staging Method; CGI-S: Clinical Global Impression-Severity; MÅDRS: Montgomery Åsberg Depression Rating Scale; QIDS-SR-16: 16-Item Quick Inventory of Depressive Symptomatology Self-Report; HAM-A: Hamilton Anxiety Rating Scale; YMRS: Young Mania Rating Scale; GAF: Global Assessment of Functioning; WSAS: Work and Social Adjustment Scale. MDQ: Mood Disorder Questionnaire; MoCA: Montreal Cognitive Assessment; CTQ: Childhood Trauma Questionnaire

*N*. MDD/BD	21/3
Age, mean (SD)	34.5 (9.69)
N. M/F	13/11
N. failed trials, mean (SD)	3.13 (1.22)
Augmentation %	74
ECT used%	17
Acute %	30
Subacute %	13
Chronic %	57
**Test**	**Mean (SD)**
MSM, mean (SD)	8.91 (2.29)
Age, mean (SD)	34.5 (9.69)
CGI	3.75 (0.94)
MÅDRS, mean (SD)	20.9 (9.8)
YMRS, mean (SD)	1.8 (2.8)
HAM-A, mean (SD)	13.25 (7.67)
MoCA	27.5 (3.7)
GAF	62.12 (10.53)
QIDS SR-16	15.01 (5.38)
STAI-A (State)	46.82 (12.04)
STAI-B (Trait)	44.11 (13.86)
WSAS	23.94 (11.21)
Ruminative response scale	58.70% (13.79)
CTQ emotional abuse	11.37 (4.39)
CTQ physical abuse	7.79 (3.89)
CTQ emotional negelct	14.21 (4.94)
CTQ physical neglect	10.21 (4.22)
CTQ sexual abuse	7.68 (5.81)

## Electronic supplementary material

Below is the link to the electronic supplementary material.

Supplementary Material 1

## Data Availability

No datasets were generated or analysed during the current study.
