# Supplementary material for: Characterisation of treatment resistant mood disorder patients in the United Arab Emirates
Source: Soc Psychiatry Psychiatr Epidemiol. 2025 Apr 3;60(9):2267–9. doi: 10.1007/s00127-025-02878-4 (PMC12378496; doi:10.1007/s00127-025-02878-4)
Supplement: Supplementary file 1 — Supplementary Material 1 [file 127_2025_2878_MOESM1_ESM.docx]

**Supplementary material**

Clinical characterization included an assessment of the level of treatment refractoriness by using the Maudsley staging method (MSM)[1]. Severity assessment included Clinical Global Impression-Severity (CGI-S) scale [2], Montgomery Åsberg Depression Rating Scale (MÅDRS) [3], The 16-Item Quick Inventory of Depressive Symptomatology Self-Report (QIDS-SR-16)[4], Hamilton rating scale (HAM-A)[5], Young Mania rating scale (YMRS)[6] and Global Assessment of Functioning (GAF)[7], Work and social adjustment scale (WSAS)[8]. Bipolar diathesis was assessed with the Mood Disorder Questionnaire (MDQ) [9], cognitive function with the Montreal Cognitive Assessment MoCA[10]. Other tools included the Childhood trauma questionnaire (CTQ)[11], and the Ruminative response scale [12].

**References**

1. Fekadu A, Donocik JG, Cleare AJ. Standardisation framework for the Maudsley staging method for treatment resistance in depression. BMC Psychiatry. 2018;18:100.

2. Guy W. ECDEU Assessment Manual for Psychopharmacology, revised. Rockville, MD, US: National Institute of Mental Health: Department of Health, Education, and Welfare Publication (ADM); 1976.

3. Montgomery SA, Asberg M. A new depression scale designed to be sensitive to change. Br J Psychiatry. 1979;134:382–389.

4. Rush AJ, Trivedi MH, Ibrahim HM, Carmody TJ, Arnow B, Klein DN, et al. The 16-Item Quick Inventory of Depressive Symptomatology (QIDS), clinician rating (QIDS-C), and self-report (QIDS-SR): a psychometric evaluation in patients with chronic major depression. Biol Psychiatry. 2003;54:573–583.

5. Hamilton M. The assessment of anxiety states by rating. Br J Med Psychol. 1959;32:50–55.

6. Young RC, Biggs JT, Ziegler VE, Meyer DA. A rating scale for mania: reliability, validity and sensitivity. Br J Psychiatry. 1978;133:429–435.

7. American Psychiatric Association. Diagnostic and Statistical Manual of Mental Disorders. Fourth Edition, Text Revision (DSM-IV-TR) [Internet]. 4th ed. Arlington, VA: American Psychiatric Association; 2000.

8. Mundt JC, Marks IM, Shear MK, Greist JH. The Work and Social Adjustment Scale: a simple measure of impairment in functioning. Br J Psychiatry. 2002;180:461–464.

9. Hirschfeld RM, Williams JB, Spitzer RL, Calabrese JR, Flynn L, Keck PE, et al. Development and validation of a screening instrument for bipolar spectrum disorder: the Mood Disorder Questionnaire. Am J Psychiatry. 2000;157:1873–1875.

10. Nasreddine ZS, Phillips NA, Bédirian V, Charbonneau S, Whitehead V, Collin I, et al. The Montreal Cognitive Assessment, MoCA: a brief screening tool for mild cognitive impairment. J Am Geriatr Soc. 2005;53:695–699.

11. Bernstein D, Fink L. Childhood Trauma Questionnaire. A Retrospective Self-Report Questionnaire and Manual. San Antonio: The Psychological Corporation; 1998.

12. Treynor W. Rumination Reconsidered: A Psychometric Analysis. Cognitive Therapy and Research. 2003;27:247–259.
